# Telepsychiatry: learning from the pandemic

**DOI:** 10.1192/bjp.2021.224

**Published:** 2022-02-18

**Authors:** Trisha Greenhalgh, Joseph Wherton

**Affiliations:** Nuffield Department of Primary Care Health Sciences, University of Oxford, Oxford OX2 6GG, UK

**Keywords:** Telepsychiatry, remote consultations, video consultations

## Abstract

This article draws on research and clinical experience to discuss how and when to use video consultations in mental health settings. The appropriateness and impact of virtual consultations are influenced by the patient’s clinical needs and social context as well as by service-level socio-technical and logistical factors.

## Introduction

Telepsychiatry is the delivery of psychiatric and mental health services through telecommunications technology, usually video. Before the pandemic, research had suggested that synchronous video consultations were safe and effective for selected patients with depression [1], anxiety [2], autism [3], psychosis [4], geriatric psychiatry [5], child and adolescent mental health needs [6], disaster response [7], as well as psychotherapy [8] and some forensic mental health uses [9]. Efforts to create guidance and systematically benchmark the quality of services had begun [10].

Outside the research setting, however, mainstream use of telepsychiatry was slow before the pandemic, limited by clinicians’ concerns around regulation, licensure and credentialing (e.g. if the clinician is seeing patients in a different country or territory), patient privacy, safety, the logistics of managing mental health crises and concerns about quality of care [11–14]. As described below, the pandemic created a strong policy push to develop and extend such services. Rapid consensus methods produced useful preliminary guidance for setting up and running in-pandemic telepsychiatry services, which were later replaced by more definitive guidance—both generic [15] and country-specific [16–18]. Many patients and clinicians had their first teleconsultation during the pandemic.

This article summarises what we have learnt to date about the place—and the challenges—of telepsychiatry as we look towards a post-pandemic future. We have structured it around the Planning and Evaluating Remote Consultation Services (PERCS) framework [19], which reminds us that sustained adoption of remote consultation services at scale will require attention to system, organisational, technology and staff domains (including policy, regulatory, logistical and staff wellbeing concerns). Even when this underpinning infrastructure is established, the question of whether a telepsychiatry consultation is appropriate for an individual patient requires a case-by-case assessment of the patient, their home and family context, their condition, and the clinical relationship. Below, we consider all these domains in turn.

## Multiple domains to consider in a telepsychiatry service

### 1 The system context: clinical need, policy push and regulatory green light

The pandemic produced a ‘burning platform’ for the introduction of telepsychiatry. High clinical need for mental health services occurred in the context of the urgent need to minimise face-to-face encounters. Relaxation of regulatory constraints [20, 21] led to a dramatic increase in the uptake of telepsychiatry models [22, 23]. This very positive system context is generally depicted as having produced, in a crisis, relatively good access to mental health services, efficient use of specialists, high patient and staff satisfaction, and smooth transitions of care [23–30]. But as the immediate crisis subsides and the system tries to move to ‘business as usual’, some patients have begun to question whether they are being short-changed with remote forms of care [31] and questions have rightly been raised about equity and digital inclusion [19]. At the time of writing, there are many unanswered questions about how regulatory and clinical governance requirements need to adapt to accommodate the effective, safe and equitable use of video and other remote modalities.

### 2 The organisational domain: workflows and the ‘virtual patient’

Clinical services which introduced remote forms of consulting ad hoc and in haste are now facing the challenge of how to align these with traditional face-to-face services in a way that supports clinical excellence and quality of care in a ‘business-as-usual’ context. Of particular relevance to mental health services is patient safeguarding and meeting ethical and regulatory standards (e.g. for undertaking and documenting informed consent, emergency management and medication prescribing) [18]. These changes may require not only reworking of clinical and administrative workflows but also changes to the risk management and governance policies that underpin them (e.g. in the processes and requirements for compulsory detention of a patient under mental health legislation).

An under-appreciated aspect of telehealth is that all clinical consultations are embedded in wider organisational routines (defined as recurring patterns of interdependent action carried out by multiple actors). The routines which support face-to-face consultations are so deeply embedded in organisational life (and in our internal mental models) that they often go unnoticed. But whether the patient is seen face-to-face or remotely, coordination has to happen to ensure that an appointment is sent, the medical record (along with test results) is made available to the clinician, the patient appears at the right time in the right waiting room, and ‘paperwork’ tasks (e.g. writing to the GP, booking a follow-up, checking test results) are completed afterwards. Considerable work is usually needed to align all these administrative routines to accommodate and sustain use of video consultations at scale.

A significant challenge in this regard is dealing with the *virtual* presence of the patient. In contrast to a face-to-face clinic, managing the patient’s ‘arrival’ at the clinic and their ‘entry’ into the consultation room, and arranging a follow-up appointment cannot be done by sending the patient to queue at different desks; these flows must be built into the system using software. Administrative systems must also be configured to distinguish between different appointment types (e.g. video, telephone, face to face) and generate appropriate documentation and communication channels. Scale-up of telepyschiatry during the pandemic required significant restructuring of patient care pathways alongside temporary suspension of regulatory constraints [21, 22].

### 3 Technologies—and the infrastructure they run on

The pandemic prompted rapid development of bespoke technologies for video consulting which were vastly more intuitive and user-friendly than earlier generations. Whilst enthusiasts may favour ‘nice-to-have’ features, as a general rule basic dependability is preferable over advanced functionality, and investment decisions for particular technologies and platforms should consider how the design relates to the capabilities (e.g. cognitive functioning, anxiety levels), preferences and digital set-up (e.g. broadband connection, data package) of both patients and clinicians.

In psychiatry and mental health contexts, most diagnostic and treatment information is gathered through talk and visual interaction. Mental health consultations are thus potentially well-suited to video technology, but set-up is important. The camera, for example, should be positioned to maximise non-verbal communication and therapeutic presence (e.g. making sure it captures face and hand expressions and avoid the need for users to concentrate on on staying ‘in view’ of one another) [32]. Clinicians and patients will need to consider how the background that is visible to the other party contributes to impression management, trust and sense of privacy. The limited view achieved on video will fail to capture all aspects of body language and behaviour (e.g. a tapping foot in an anxious patient).

Video and audio connection must be sufficiently high-quality to ensure that expressions are visible and conversation flows without too much lag [33]. Minor technical breakdowns (e.g. difficulty establishing audio connection or temporary freezing of the video) tend not to disrupt the clinical interaction as they are typically easy to resolve so long as both parties have adequate technical skills (but can be prohibitive if they do not) [33]. Contingency plans are needed in case of technical failures (e.g. agreeing a backchannel, such as telephone, in case of cut-out and plans for dealing with patient anxiety).

Technologies are rarely plug-and-play; they require infrastructure including a physical scaffolding (hardware and software, as well as buildings, wires, connections, clinical record templates, charts and so on), people (the individuals whose roles and interactions make the service possible and the training and oversight of those individuals), and the standards and guidance needed for the system to work effectively, safely and legally. Efforts to implement and spread remote consultation services often fail or stall due to problems interfacing the new technology with local material constraints (e.g. physical space), legacy computer systems, patterns of working, and historically-established standards [34].

### 4 The staff domain: acceptance, well-being, training

Most technologies in healthcare fail because clinicians do not use them. The research literature shows that clinicians are overwhelmingly driven by standards of professional excellence, and the main reason why they fail to adopt technologies (or adopt but soon abandon them) is concern about potential compromises to the quality and safety of care [35]. Training clinicians to use video technologies is important—but if widespread and sustained uptake and use of telepsychiatry is the goal, careful attention must also be paid to professional concerns about the quality of the consultation (e.g. the need to see the whole patient not just their head and torso), risk and safety, confidentiality, and equity [19]. These concerns must be considered both at the level of clinical guidelines (which can give broad indications for when telepsychiatry is more or less suitable) and on an individual, case-by-case basis (see examples below). Some staff may prefer to work remotely (e.g. if they are clinically vulnerable themselves). Others—particularly less experienced clinicians—may become stressed, burnt out and demoralised, partly because remote consultations are more cognitively demanding and partly because they may have fewer opportunities for the clinical training and mentoring they need. Hence, the policy push to expand telepsychiatry for reasons of ‘efficiency’ must be tempered by the needs, concerns and preferences of the workforce.

### 5 The reason for consulting

Whilst some clinical conditions lend themselves to video format more than others, every patient is different and there are few if any absolute contraindications to video consulting. Box 1 gives some fictional cases to illustrate how the assessment of the clinical reason for consulting does not *determine* the optimum modality. Rather, the clinical need(s) must be assessed in the light of patient, home and family factors and the nature of (and need for) the therapeutic relationship, which are considered in the next sections.

In Case 1, a video consultation for this patient with autism seems appropriate, for several reasons. The patient is already digitally literate and his home has a suitable broadband connection and computer hardware. He has previously expressed a preference for remote consultations and has experience of these. Unlike some teenagers, he has a private space from which to connect and his parents have a track record of respecting his privacy during his medical appointments. A trained clinician has established that he is not in a high-risk category.

In Case 2, there are clinical, social and technical reasons why a video consultation may not be the best choice. As the GP has discovered, suspected mania is not easily assessed by telephone. A video connection would allow visual assessment of the patient’s demeanour and behaviour, allowing a more confident diagnosis, but she is uncooperative and unlikely to engage. From the history, she may require legal detention measures. She is likely to require a change in medication but it is not clear how this would be supplied to her. The family’s digital set-up is limited and data poverty mean they will not be comfortable with the lengthy consultation that is likely needed, and the encounter may be thwarted by poor technical connection.

Case 3 illustrates the complex challenges of institutionalised psychogeriatric patients. This patient clearly needs a full clinical and psychiatric assessment as well as a medication review. Whereas in the previous cases, the overall picture points clearly in favour (Case 1) and against (Case 2) attempting a video consultation, in this case an emergent approach may be needed (e.g. discuss the option of video with staff who know Daniel and take their views into account). It may be that a video consultation could be attempted as a first step, but extended to a face-to-face assessment if it proves clinically, socially or technically inadequate.

### 6 The patient: capacity, capability, comorbidities, preferences

Whilst guidance now exists on the principles of safe and effective telepsychiatry [15–18], and provision in practice will inevitably be constrained by what services are available locally and what capacity exists in those services, the decision as to whether a particular patient should be seen remotely or face-to-face necessarily involves judgement. The decision should take account of the patient’s capabilities and capacity (e.g. English fluency, sensory or cognitive impairment, capacity to consent) [16, 18] as well as their comorbidities, and consider how all these may influence contingency plans (safety-netting) and other risk management strategies. Unless there are over-riding reasons not to, patients should be given a choice so they can select their preferred format. Careful consideration must be paid to ‘high risk’ issues (e.g. risk of violence, aggression or self-harm, stability of the patient’s condition, and intoxication).

### 7 The home and family: support, structural challenges and digital inclusion

Consulting from home may be possible and preferred—but the patient may not have a home. There may be physical limitations (e.g. lack of private, quiet space), technical ones (lack of digital technologies or the infrastructure to run them), or psychosocial ones (distraction, coercion, violence). Mental health patients may experience multiple jeopardy from (for example) poverty, poor housing, low health literacy, weak social networks, psychological stress (e.g. from fear of crime) and language and cultural discordance. To these we must now add digital inequalities, defined as differential access to healthcare depending on digital access, digital literacy or both [36]. It is important to go beyond a binary perspective (presence or absence of Internet access) when assessing digital inclusion and consider how much bandwidth, data, IT literacy and skills, and power (e.g. over who in the household has use of the computer or smartphone) people have. For patients whose home set-up does not allow safe video consulting, non-digital alternatives (the option to ask for a traditional face-to-face appointment) and flexibility in how remote is used (e.g. allowing patients to consult with the video switched off if they prefer) are important components of a digital inclusion strategy. In some settings, local health or care services can provide a private space or ‘pod’ from which a patient can arrange to connect to their video appointment.

### 8 The clinical relationship

Much (though perhaps not all) mental health consultations benefit from a strong therapeutic alliance. Some authors have argued that the therapeutic alliance achieved via video during the pandemic was comparable to that in in-person encounters (video can be seen as a vehicle for building rapport and trust rather than an obstacle to achieving it) [37]. For instance, video may allow the clinician to witness some of the living circumstances the patient describes in their sessions, provide a comfortable space to engage in relaxation exercises, and facilitate engagement and playful activities with children. The video format can even provide a preferred format for the therapeutic alliance—for example for those experiencing mood disorders and interpersonal avoidance who may find close contact overwhelming [37]. But this is contingent on the capabilities of the clinician to account for the physical and symbolic differences in the technology-supported environment, and to make adjustments to convey empathy and warmth.

Our previous research highlighted the ‘opening’ to be an important part of the consultation because this is when both patient and clinician establish whether the video and/or audio connection is adequate before proceeding with the consultation proper. Greetings and rapport-building should be used to help put patients at ease, given that more conventional forms of prosocial interaction and contact during face-to-face medical encounters (eg, shaking hands and inviting into the consultation room) are absent.

Facial expressions and hand gestures can help compensate for loss of physical presence and body language. Both clinicians and patients will also need to deal with inherent problems of latency (time delay in transmission from one end of the call to the other), especially as responsiveness to what the other person is saying is essential for conveying empathy and understanding. Clinicians should attend to effective turn-taking—for example, using longer pauses to minimise overlap and inviting the patient to speak [38].

## Conclusion

Whilst telepsychiatry is not a panacea, there is good reason to be optimistic about its potential in most though not all patients and settings. There will, inevitably, be a personal and an organisational learning curve before people become confident in using this new medium for clinical encounters. It will be important to assess both patient and staff satisfaction and comfort with telepsychiatry models over time, as increasing familiarity may lead to increased confidence and acceptance.

A major growth area for telepsychiatry in the next few years is likely to be refinement of the draft professional guidance, competences and quality standards that have been produced to date [10, 15, 18]. It is important that practitioners harness and share knowledge on effective approaches through communities of practice, produce rules of thumb on what is generally safe, and engage with professional bodies and defence societies to develop contemporary definitions of good clinical practice.

## Figures and Tables

**Figure 1:**
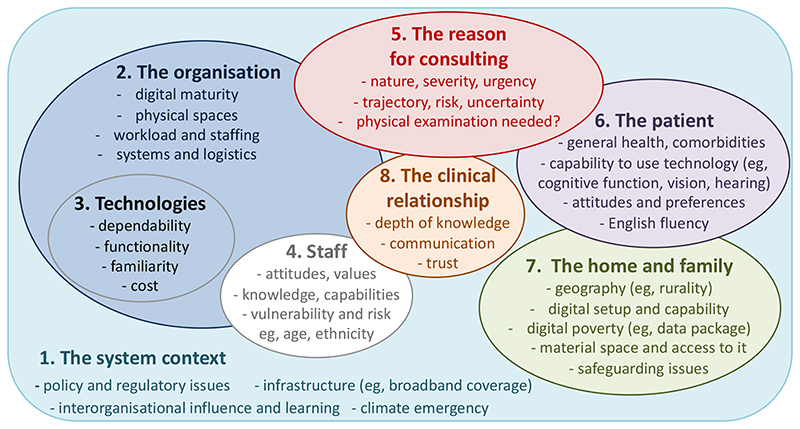
The PERCS (Planning and Evaluating Remote Consultation Services) framework Adapted under Creative Commons Licence from [19]

## Data Availability

Not applicable (all data are in the public domain as this is a review).

## Box 1 Three clinical cases

### Case 1: A teenager with autism

Robert is a 15-year-old boy with high-functioning autism; he is well known to the Child and Adolescent Mental Health Services. Before the pandemic, he attended mainstream school and had been working towards his GCSE exams. He enjoys playing computer games in his bedroom and is adept at programming. Because he disliked attending the hospital (he found it noisy and disorienting), he had been seeing his community mental health nurse via video consultation every three months. With prolonged lockdown, his routines have been disrupted and he has become depressed. His mother is alarmed that he has begun to self-harm, though at present this is limited to superficial cutting. His community psychiatric nurse is confident that he is not suicidal but feels he should see a consultant for full assessment of his mental state and possible prescription of medication.

### Case 2: A patient with a possible manic episode

Reena is a 37-year-old waitress who lives in a remote rural setting; she has been under the care of her GP for several years for mood swings. She has had several episodes of moderate depression, managed by her GP with talking therapy and medication. On this occasion, Reena’s husband contacted the GP saying she had become agitated over the past week. The GP had tried to speak to her on the phone but she was unable to continue a conversation. Her husband noted that she was pacing constantly and sleeping only 2-3 hours per night, and had on one occasion left the house in her underwear and had to be brought back by a neighbour. He had returned form work yesterday to find her smelling of alcohol and the children unfed. The family live in rented council accommodation and whilst they have a broadband connection, the husband says their data package is somewhat restrictive so he hopes that the video consultation won’t last more than a few minutes. Reena has never used the family computer, which was bought 10 years ago and has a habit of crashing.

### Case 3: A psychogeriatric patient

Daniel is a 76-year-old retired engineer who has been living in a residential home for two years. He has diabetes, heart failure, depression, a leg ulcer and gout, as well as progressively worsening cognitive function (perhaps early Alzheimer’s disease). He has recently become incontinent of urine (though a specimen showed no growth), and seems to be becoming slower and more withdrawn. His medication includes sertraline, insulin injections, allopurinol, enalapril, and omeprazole. He has begun to decline all his tablets (though he will take them with coaxing) and this morning refused to have his insulin injection. The care home staff have asked for an urgent assessment. The care home is well connected digitally and staff are used to supporting their clients to have video conversations with their relatives and with clinicians. Daniel is chair-bound so he may co-operate to some extent with a video consultation, but he may not understand that the person on the screen is a doctor.
